# A Novel Role For Nanog As An Early Cancer Risk Marker In Patients With Laryngeal Precancerous Lesions

**DOI:** 10.1038/s41598-017-11709-9

**Published:** 2017-09-11

**Authors:** Juan P. Rodrigo, M. Ángeles Villaronga, Sofía T. Menéndez, Francisco Hermida-Prado, Miquel Quer, Isabel Vilaseca, Eva Allonca, Daniel Pedregal Mallo, Aurora Astudillo, Juana M. García-Pedrero

**Affiliations:** 1 Department of Otolaryngology, Hospital Universitario Central de Asturias and Instituto Universitario de Oncología del Principado de Asturias, Oviedo, CIBERONC Spain; 2 Department of Otolaryngology, Hospital Santa Creu i Sant Pau, Barcelona, Spain; 3 Department of Otolaryngology, Hospital Clínic, Barcelona, Spain; 4 Department of Pathology, Hospital Universitario Central de Asturias, Oviedo, Spain

## Abstract

NANOG is a master regulator of embryonic stem cell pluripotency, found to be frequently aberrantly expressed in a variety of cancers, including laryngeal carcinomas. This study investigates for the first time the role of NANOG expression in early stages of laryngeal tumourigenesis and its potential utility as cancer risk marker. NANOG protein expression was evaluated by immunohistochemistry using two large independent cohorts of patients with laryngeal precancerous lesions, and correlated with clinicopathological parameters and laryngeal cancer risk. NANOG expression was detected by immunohistochemistry in 49 (60%) of 82 laryngeal dysplasias, whereas expression was negligible in patient-matched normal epithelia. Strong NANOG expression was found in 22 (27%) lesions and was established as cut-off point, showing the most robust association with laryngeal cancer risk (*P* = 0.003) superior to the histological classification (*P* = 0.320) the current gold standard in the clinical practice. Similar trends were obtained using a multicenter validation cohort of 86 patients with laryngeal dysplasia. Our findings uncover a novel role for NANOG expression in laryngeal tumourigenesis, and its unprecedented application as biomarker for cancer risk assessment.

## Introduction

NANOG is a key transcription factor critical for the acquisition and maintenance of both embryonic and induced pluripotency^1, 2^. In addition to its critical role during embryogenesis^3, 4^, it has been recently demonstrated that NANOG exerts a lineage-restricted mitogenic function in stratified epithelia in adult tissues^5^. Inducible ubiquitous overexpression of NANOG in mice selectively caused hyperplasia in stratified epithelia, as well as increased proliferation and aneuploidy. In addition, NANOG overexpression in mouse skin epithelia was found to favour malignant transformation of skin papillomas induced by chemical carcinogenesis, thus providing an *in vivo* evidence for the oncogenic role of NANOG in squamous cell carcinomas^6^. Furthermore, NANOG has been found frequently aberrantly expressed in a variety of human cancers, including head and neck squamous cell carcinomas (HNSCC)^6, 7^. NANOG expression in cancer has been associated with chemoresistance^8, 9^, epithelial to mesenchymal transition (EMT)^6^ and poor clinical outcome^7, 10–12^.

Nevertheless, the role of NANOG in the early stages of HNSCC tumourigenesis and its possible implication in malignant transformation and acquisition of an invasive phenotype remains to be determined.

This prompted us to investigate NANOG protein expression in laryngeal tumourigenesis using a large series of 82 laryngeal precancerous lesions to establish correlations with clinicopathological parameters and the risk of progression to invasive carcinoma. This work unveils the clinical utility of NANOG expression as cancer risk marker in patients with laryngeal dysplasias, showing superior predictive power to histology. These results were further confirmed using an independent multicenter cohort of 86 patients with laryngeal premalignancies.

## Results

### NANOG protein expression in the early stages of laryngeal tumorigenesis

**Figure 1:**
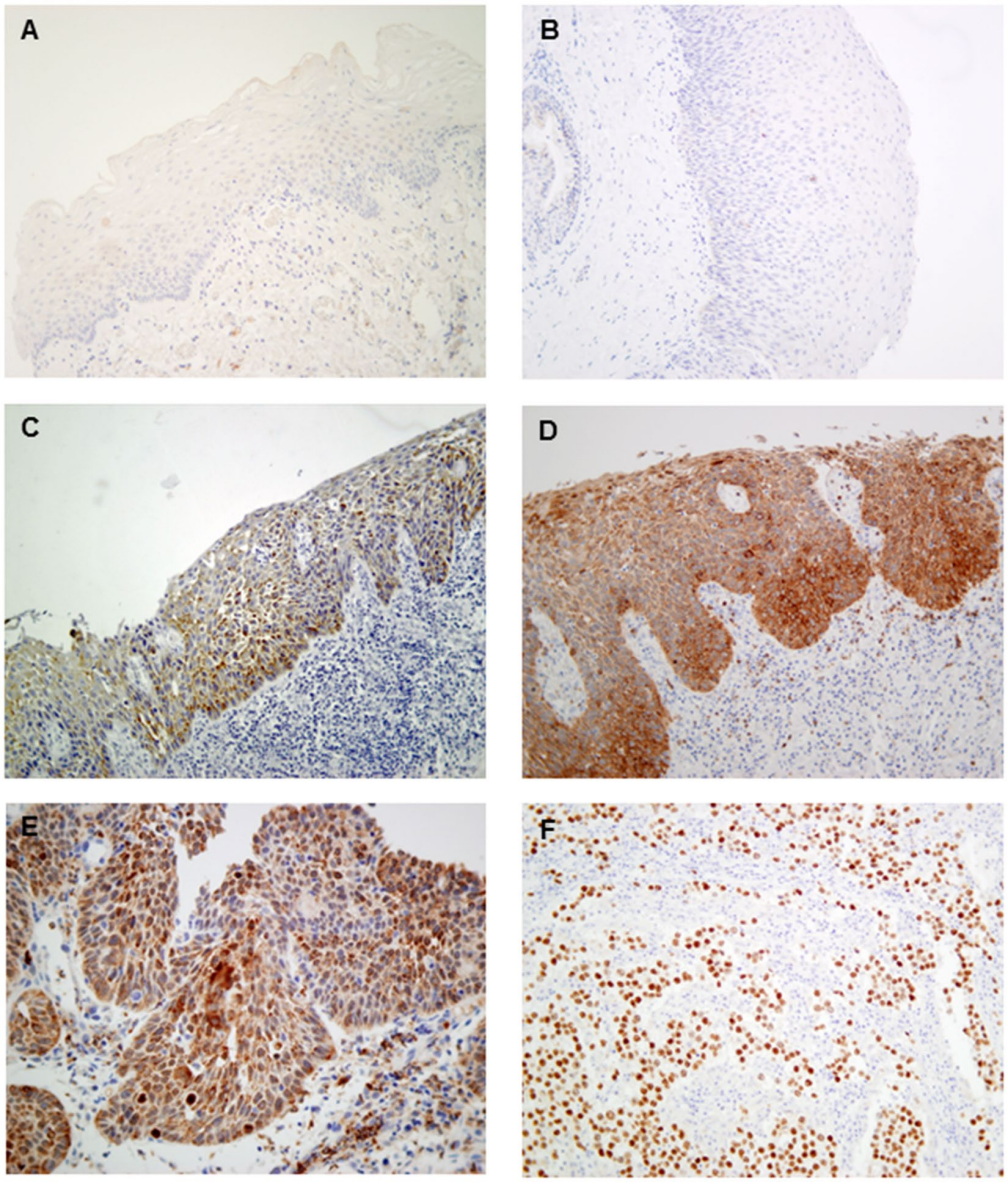
Immunohistochemical analysis of NANOG expression in laryngeal dysplasias. Normal adjacent epithelia showed negative staining (**A**). Representative examples of low-grade dysplasias showing negative NANOG staining, score 0 (**B**) and cytoplasmic NANOG staining score 1 (**C**), a high-grade dysplasia with cytoplasmic NANOG staining score 2 (**D**), and a high-grade dysplasia with nuclear NANOG staining (**E**). Human seminoma was used as positive control, showing strong nuclear NANOG staining (**F**). Original magnification ×200 (E ×400).

Immunohistochemical analysis of NANOG protein expression was performed on a set of 82 laryngeal dysplasias. Positive NANOG expression was detected in the cytoplasm in 49 (60%) dysplasias (scored as 1 and 2), whereas expression was negligible in both stromal cells and normal adjacent epithelia (Fig. 1). Twenty-two (27%) lesions showed strong NANOG expression (score 2).

Cytoplasmic NANOG protein expression did not correlate with the histopathological classification: 9 (64%) of the 14 lesions with low-grade dysplasia, and 40 (59%) of the 68 lesions with high-grade dysplasia exhibited cytoplasmic NANOG protein expression (Chi-square *P* = 0.930). Strong NANOG expression was found in 4 (29%) low-grade dysplasias, and 18 (26%) high-grade dysplasias (Fisher’s exact *P* = 1.000). In addition, nuclear NANOG expression was detected in 7 dysplasias that also showed cytoplasmic expression: one case with weak and 6 cases with strong cytoplasmic expression (*P* = 0.001, Pearson ρ = 0.375).

### Associations with laryngeal cancer risk

During the follow-up period, 24 (29%) of 82 patients developed an invasive carcinoma at the same site of the previous premalignant lesion. The mean time to cancer diagnosis in the cases that progressed was 28 months (range 6 to 66 months). No significant differences attributable to age were observed (*P* = 0.703) between the group of patients who developed cancer (mean, 65 years) and those who did not (mean, 64 years). The mean tobacco consumption for patients who developed an invasive carcinoma was 58.6 packs-year, compared to 52.7 packs-year for those who did not develop cancer (*P* = 0.316). Similarly, no significant differences in laryngeal cancer risk were observed between the subgroup of patients who continued smoking (14 patients) and those who ceased smoking (*P* = 0.757).

There was no statistically significant correlation in this cohort between the histopathological grade and the risk of progression to laryngeal cancer (*P* = 0.748; Table 1), although high-grade dysplasias showed a higher cancer risk (HR = 1.517, 95% CI 0.452 to 5.089; *P* = 0.499; Table 2).

**Table 1:** Evolution of the premalignant lesions in relation to histopathological diagnosis and NANOG protein expression.

Characteristic	No. of cases (%)	Progression to carcinoma (%)	*P*
**Histopathological diagnosis**			
Low-grade dysplasia	14(17)	3(21)	0.748^†^
High-grade dysplasia	68(83)	21(31)	
**Cytoplasmic NANOG protein scores**			
Negative (score 0)	33(40)	6(18)	0.009^#^
Weak/Moderate (score 1)	27(33)	6(22)	
Strong (score 2)	22(27)	12(55)	
**Cytoplasmic NANOG protein expression**			
Negative to Moderate	60(73)	12(20)	0.005^†^
Strong (score 2)	22(27)	12(55)	
**Nuclear NANOG protein expression**			
Negative	75(91)	20(27)	0.186^†^
Positive	7(9)	4(57)	

^#^Chi-square and ^**†**^ Fisher’s exact tests.

**Table 2:** Univariate Cox Proportional Hazards Model to Estimate Laryngeal Cancer Risk in the exploratory cohort.

Characteristic	*P*	Hazard Ratio	95% CI
-Age (above *vs* below the mean)	0.652	1.203	0.539 to 2.686
-Smoking (above *vs* below the mean)	0.41	1.4	0.629 to 3.119
-Histology (High-grade *vs* low-grade dysplasia)	0.499	1.517	0.452 to 5.089
-Cytoplasmic NANOG expression (Score 2 *vs* 0–1)	0.003	1.826	1.222 to 2.728
-Nuclear NANOG expression	0.063	2.773	0.945 to 8.134

**Figure 2:**
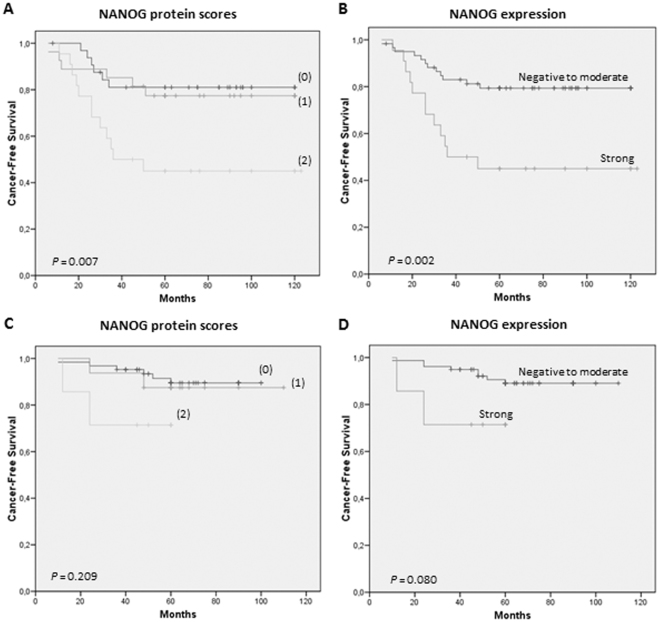
Kaplan-Meier cancer-free survival curves in the exploratory series of 82 patients with laryngeal dysplasias categorized by NANOG protein scores (**A**) or NANOG protein expression dichotomized as strong (score 2) *versus* negative to moderate expression (scores 0–1) (**B**). Kaplan-Meier cancer-free survival curves in the validation series of 86 patients with laryngeal dysplasias categorized by NANOG protein scores (**C**) or NANOG protein expression dichotomized as strong (score 2) *versus* negative to moderate expression (scores 0–1) (**D**). *P* values were estimated using the log-rank test.

Interestingly, we found that cytoplasmic NANOG protein scores significantly correlated with an increased laryngeal cancer risk (Table 1, and Fig. 2A, log-rank *P* = 0.007). Therefore, strong cytoplasmic NANOG expression (score 2) was used as a cut-off point in our subsequent analyses (Table 2, *P* = 0.003, and Fig. 2B, log-rank *P* = 0.002). Patients carrying strong cytoplasmic NANOG-expressing lesions (score 2) experienced a significantly higher progression to laryngeal cancer than those with absent to moderate (scores 0–1) expression (HR = 1.826; 95%CI, 1.222–2.728; *P* = 0.003). At 5 years after the patients were diagnosed, 55% of the patients with strong cytoplasmic NANOG expression developed laryngeal cancer compared with 20% of the patients with negative to moderate NANOG expression (Fisher’s exact test, *P* = 0.005; Table 1). Nuclear NANOG expression also tended to associate with increased laryngeal cancer risk (Fisher’s exact test, *P* = 0.186; Table 1).

Univariate Cox analysis showed that strong cytoplasmic NANOG expression was the only significant predictor of laryngeal cancer risk (Table 2). The sensitivity, specificity, positive predictive value (PPV), and negative predictive value (NPV) of cytoplasmic NANOG expression were 50%, 83%, 54%, and 80%, respectively.

### Multicenter validation of NANOG as cancer risk marker

To further confirm these results, immunohistochemical analysis of NANOG expression was also carried out in an independent series of 86 laryngeal premalignancies from two different collaborating institutions in Barcelona, Spain (Hospital de la Santa Creu i Sant Pau and Hospital Clínic). Cytoplasmic NANOG expression (scored as 1 and 2) was detected in 23 (27%) laryngeal dysplasias, and 7 (8%) lesions showed strong cytoplasmic NANOG expression. Nuclear NANOG expression was detected in nine of the cases with cytoplasmic expression; in 5 cases with weak and in 4 cases with strong cytoplasmic expression (*P* < 0.001, Pearson ρ = 0.6).

The proportion of cases with strong cytoplasmic expression was considerably lower than that observed in our exploratory series of laryngeal dysplasias from the Hospital Universitario Central de Asturias. However, noteworthy the proportion of patients who developed an invasive carcinoma in the validation series was also notably low. Thus, during the follow-up period 10 (12%) patients developed an invasive carcinoma at the same biopsy site. More importantly, the results obtained in the validation cohort confirmed the relationship of NANOG expression with laryngeal cancer risk (Fig. 2C,D). Similarly, strong cytoplasmic NANOG expression showed the most robust association with laryngeal cancer risk, although the differences did not reach statistical significance, probably due to the small proportion of cases that progressed to cancer (log-rank *P* = 0.080; Fig. 2D), whereas histology was not a significant predictor of cancer development (log-rank *P* = 0.274). Nuclear NANOG expression was not associated with laryngeal cancer risk in this cohort (*P* = 0.59).

Also in this cohort, univariate Cox analysis showed that strong cytoplasmic NANOG expression was the better predictor of laryngeal cancer risk (Table 3). The sensitivity, specificity, positive predictive value (PPV), and negative predictive value (NPV) of cytoplasmic NANOG expression were 20%, 93%, 29%, and 90%, respectively.

**Table 3:** Univariate Cox Proportional Hazards Model to Estimate Laryngeal Cancer Risk in the validation cohort.

Characteristic	*P*	Hazard Ratio	95% CI
-Age (above *vs* below the mean)	0.826	1.149	0.332 to 3.973
-Smoking (above *vs* below the mean)	0.246	2.177	0.584 to 8.113
-Histology (High-grade *vs* low-grade dysplasia)	0.286	0.502	0.142 to 1.779
-Cytoplasmic NANOG expression (Score 2 *vs* 0-1)	0.1	1.906	0.876 to 4.148
-Nuclear NANOG expression	0.475	0.042	0.000 to 256.12

## Discussion

We recently reported that NANOG is frequently aberrantly expressed in squamous cell carcinomas, including HNSCC^5^. In addition, evidence was also provided to support that NANOG plays an active role *in vivo*, promoting malignant conversion of skin papillomas into carcinomas in transgenic mice^6^. To further and significantly extend these data, this study investigates for the first time NANOG expression in early stages of laryngeal tumourigenesis to uncover its role in malignant transformation and potential clinical application as cancer risk marker.

Using two large independent cohorts of patients with laryngeal precancerous lesions, we consistently demonstrate that NANOG is frequently abnormally expressed in laryngeal dysplasias but not in normal adjacent epithelia. More importantly, strong cytoplasmic NANOG protein expression (score 2) significantly correlated with higher cancer incidence in both patient cohorts. It is also worth noting that strong cytoplasmic NANOG expression showed the most robust association with laryngeal cancer risk and superior predictive power than the histological classification, which remains the current gold standard in the clinical practice. Although the sensitivity of cytoplasmic NANOG expression to detect patients who will develop laryngeal cancer was not high (50% and 20% in the exploratory and the validation cohort, respectively), it consistently showed to be highly specific in the two independent cohorts analysed (83% and 93% in the exploratory and validation cohorts, respectively). Hence, this biomarker test is characterised by high negative predictive values and could therefore be helpful to identify patients at low probability of progression to laryngeal cancer. Consequently, clinical assessment of NANOG staining could be useful for ruling out the disease.

Regarding the differences in progression risk between the two patient cohorts, the higher laryngeal cancer risk observed in our patient series from Asturias may be attributed to demographic differences, since Asturias is the region with the highest laryngeal cancer incidence in Spain and also one of the highest in the world^13^. Consistent with this notion, the proportion of patients carrying lesions with strong NANOG expression was also higher in the exploratory cohort from Asturias, probably underlying that this population harbours more genetic and molecular alterations due to higher carcinogen exposure (as reflected by the higher tobacco consumption rates), which would ultimately provide an additional advantage for tumour formation.

Like other epithelial cancers, laryngeal carcinogenesis appears to evolve through a multistep process that involves biomolecular changes caused by carcinogen exposure, ensuing premalignant lesions and consequent invasive carcinoma^14, 15^. Although lesions with dysplastic features are thought to be at a higher cancer risk^16^, some cancers develop from lesions lacking dysplastic changes. Additional objective and reliable markers are therefore needed to identify more accurately high-risk lesions beyond current clinical and histopathological criteria^17^, which will undoubtedly help the clinicians to choose the most adequate therapeutic option. Since immunohistochemical analysis of NANOG is relatively simple and easy to interpret, it seems reasonable to recommend this molecular test to be included as complementary marker for cancer risk assessment and decision-making. Nevertheless, routine implementation will require further confirmation of these results in large prospective studies.

Therefore, cytoplasmic NANOG expression emerges as a clinically and biologically relevant feature in early stages of laryngeal tumourigenesis that contributes to malignant transformation and laryngeal cancer development. Aberrant NANOG expression appears to be a distinctive characteristic in human squamous cell carcinomas^5^, it has been associated to chemoresistance and poor disease outcome in various cancers^7–12, 18^, and according to the herein presented data, it also plays a prominent role in early stages of tumourigenesis and malignant transformation. NANOG immunostaining is usually detected in the nuclei of pluripotent cells, as shown in our seminoma control. Nuclear NANOG expression was also detected in laryngeal dysplasias; however, cytoplasmic NANOG expression was the most predominantly found and clinically relevant.

Our findings are in good agreement with previous reports that showed NANOG expression in the cytoplasm of tumour cells, associated with poor prognosis^5, 11^. Thus, cytoplasmic NANOG expression has been frequently detected in HNSCC tissue specimens^5^. Cytoplasmic NANOG expression has also been frequently detected in high-grade OSCC^11^. In addition, patients with strong NANOG expression exhibited significantly lower overall survival rates than those with weak or negative NANOG expression, suggesting that OSCCs with high cytoplasmic NANOG expression may possess aggressive characteristics, and ultimately poor prognosis^11^. In nasopharyngeal carcinomas, cytoplasmic NANOG was particularly evident at the invasive edge of tumours compared to low cytoplasmic NANOG expression in the non-cancerous epithelium, and showed clinical relevance associated to poor prognosis^19^. Similarly, cytoplasmic NANOG-positive stromal cells promoted human cervical cancer progression, with mesenchymal stem cells (MSCs) being one type of cytoplasmic NANOG-positive cells in cervical cancer stroma that participate in the progression of cervical cancer both *in vitro* and *in vivo* ^20^.

The oncogenic role of NANOG involves transcriptional induction of an EMT program and stem cell features^6, 18^, leading to sustained unlimited growth, increased cell migration and acquisition of invasive phenotype. Hence, these data reflect that NANOG may represent a promising therapeutic target in the prevention and treatment of epithelial cancers.

Together our findings uncover a novel role for NANOG expression in laryngeal tumourigenesis, and its unprecedented clinical application as biomarker for cancer risk assessment in patients with precancerous lesions.

## Methods

### Patients and tissue specimens

Surgical tissue specimens from patients who were diagnosed of laryngeal dysplasia at the Hospital Universitario Central de Asturias between 1996 and 2010 were retrospectively collected, in accordance to approved institutional review board guidelines. All experimental protocols were approved by the Institutional Ethics Committee of the Hospital Universitario Central de Asturias and by the Regional CEIC from Principado de Asturias (approval number: 81/2013 for the project PI13/00259). Informed consent was obtained from all patients. Patients must meet the following criteria to be included in the study: *i*) pathological diagnosis of laryngeal dysplasia; *ii)* with lesions of the vocal folds *iii)* no previous history of head and neck cancer; *iv)* complete excisional biopsy of the lesion; *v)* a minimum follow-up of five years (or until progression to malignancy occurred); and *vi)* patients with a diagnosis of laryngeal dysplasia who developed cancer within the next six months were excluded from the study. Eighty-two consecutive patients who met these criteria were included in this study. All the patients were treated with macroscopically complete excisional biopsy of the lesion, either with CO_2_ laser or with cold instruments. Microscopically surgical margins were not assessed. No other treatments were administered. Patients were followed up every two months in the first six months after completing the treatment, every three months until the second year, and every six months thereafter.

Representative tissue sections from the original biopsy material were obtained from archival, paraffin embedded blocks and the histological diagnosis and epithelial dysplasia grade was confirmed in all the cases by an experienced pathologist (A.A.). The sections selected for study also contained normal epithelia as internal controls. After review, the premalignant lesions were classified into the categories of low-grade dysplasia (14 cases, 17%), and high-grade dysplasia (68 cases, 83%) following the WHO classification 4th Edition^21^.

All patients were men, with a mean age of 64 years (range 36-86 years). All but two patients were smokers, 44 (54%) moderate (1-50 pack-year) and 36 (44%) heavy (>50 pack-year) smokers. The mean tobacco consumption was 54 pack-year (range 0-150). After the diagnosis, all the patients who were active smokers received smoking cessation advice; however, 14 of them continued smoking.

In addition, an independent cohort of 86 patients with a diagnosis of laryngeal dysplasia was used as a validation series. This series included consecutive patients treated at two collaborating institutions in Barcelona (Hospital de la Santa Creu i Sant Pau and Hospital Clinic) between 1996 and 2010, who met the same inclusion criteria above-described for the exploratory series. Surgical tissue specimens were retrospectively collected, with the approval of their institutional review boards and in accordance to ethical and legal protection guidelines. The histological diagnosis and epithelial dysplasia grade was verified in all the cases by the same pathologist at our institution (A.A.), and after review, the premalignant lesions were classified into low-grade (22 cases, 26%), and high-grade dysplasia (64 cases, 74%) according to the WHO classification 4^th^ Edition^19^.

All but seven patients were men, with a mean age of 62 years (range 30–87 years). All but nine patients were smokers, 47 (55%) moderate (1–50 pack-year) and 30 (35%) heavy (>50 pack-year) smokers. The mean tobacco consumption was 42 pack-year (range 0–120).

### Immunohistochemistry

The formalin-fixed, paraffin-embedded tissues were cut into 3-µm sections and dried on Flex IHC microscope slides (Dako). The sections were deparaffinized with standard xylene and hydrated through graded alcohols into water. Antigen retrieval was performed using Envision Flex Target Retrieval solution, high pH (Dako). Staining was done at room temperature on an automatic staining workstation (Dako Autostainer Plus) with Nanog (D73G4) XP® Rabbit monoclonal antibody (Cell Signaling technology, Inc.) at 1:200 dilution using the Dako EnVision Flex + Visualization System (Dako Autostainer). Counterstaining with haematoxylin was the final step.

A semiquantitative scoring system based on staining intensity was applied, as previously reported^5^. Immunostaining of the dysplastic areas was scored blinded to clinical data by two independent observers as negative (absence of staining, score 0), weak to moderate (some cytoplasmic staining in dysplastic areas, score 1), and strong protein expression (intense and homogeneous cytoplasmic staining in dysplastic areas, score 2), with a high level of inter-observer concordance (>95%). As in some cases nuclear staining was observed, the cases were also scored as positive/negative based on the presence of nuclear staining in dysplastic areas. Human seminoma was used as positive control, showing strong nuclear NANOG staining.

### Statistical analysis

χ^2^ and Fisher’s exact tests were used for comparison between categorical variables. For time-to-event analysis, Kaplan-Meier curves were plotted. Differences between survival times were analysed by the log-rank method. Cox proportional hazards models were utilized for univariate analysis. The hazard ratios (HR) with 95% confidence interval (CI) and P values were reported. All tests were two-sided. p values of ≤0.05 were considered statistically significant.
